# Effect of Gypsum on Hydration and Hardening Properties of Alite Modified Calcium Sulfoaluminate Cement

**DOI:** 10.3390/ma12193131

**Published:** 2019-09-25

**Authors:** Pei Li, Zhiqiang Ma, Zhong Zhang, Xumin Li, Xiaolei Lu, Pengkun Hou, Peng Du

**Affiliations:** Shandong Provincial Key Laboratory of Preparation and Measurement of Building Materials, University of Jinan, Jinan 250022, China; lipei0418@163.com (P.L.); Mazq6100@163.com (Z.M.); 15254152857@163.com (Z.Z.); 17862911266@163.com (X.L.); mse_luxl@ujn.edu.cn (X.L.); mse_houpk@ujn.edu.cn (P.H.)

**Keywords:** gypsum, alite modified calcium sulfoaluminate cement, setting time, hydration, compressive strength

## Abstract

Calcium sulphoaluminate cement (CSA) has the characteristics of quick hardening, high early strength and high impermeability, however its strength growth persistence in the middle and late stages (after the age of 3 days) is poor. In order to improve this disadvantage, the pilot production of alite (C_3_S) modified CSA (AMCSA) clinker was carried out by liquid phase manipulation and barium ion doping technology. The effects of different dosages of gypsum on the hydration and hardening properties of AMCSA, such as setting time, hydration rate, compressive strength and hydration products, were studied. The results show that the mineral content of ye’elimite, C_2_S, C_3_S and iron phase in the calcined AMCSA clinker are 48.5 wt.%, 32.6 wt.%, 11.7 wt.% and 7.2 wt.% respectively, which are close to the designed mineral composition. The stable coexistence of ye’elimite and C_3_S in the same clinker system is realized. The initial and final setting time of AMCSA are retarded with the increasing gypsum dosage. When the gypsum dosage is 15 wt.% under the experimental conditions in this study, the AMCSA mortar reaches the highest compressive strength at every age. The strength of AMCSA mortar at 28 days is still significantly improved compared with that at 3 days, which indicates that the shortcoming of the low strength growth persistence of CSA in the middle and late stages is improved.

## 1. Introduction

Calcium sulfoaluminate cement (CSA) exhibits the characteristics of low calcining temperature [1,2], quick hardening, high early strength and high impermeability [3,4,5], therefore it is considered to have huge potential as a special engineering material, and is widely used in special engineering, such as winter construction, rapid repair protection engineering and ocean engineering [6,7]. However, one of the fatal disadvantages limiting CSA application is the low strength growth persistence in the middle and late stages, and it can not be easily improved [8]. This is mainly because C_4_A_3_$\bar{S}$, the main mineral of CSA, is an early-strength mineral [9,10], and its strength in the middle and late stages (after the age of 3 days) is almost not developed. C_2_S is another important mineral in CSA, where the strength increases very slowly and the hydration degree is only 20% at 28 days [11], so its support for the strength growth persistence is also minimal. These defects need to be improved by the fundamental design angle of the cement clinker. Therefore, this disadvantage should be improved from the perspective of cement clinker mineral composition design.

C_3_S is one of the main minerals of Portland cement, which has a persistent strength growth. The hydration of C_3_S and C_4_A_3_$\bar{S}$ can promote each other, which can manipulate the proportion of hydration products [12] and increase the absolute value of strength. Therefore, some scholars proposed that C_3_S and C_4_A_3_$\bar{S}$ coexist in the same clinker system to complement each other [13,14]. But it is difficult to realize the coexistence of C_3_S and C_4_A_3_$\bar{S}$ due to their contradictory formation temperature. C_4_A_3_$\bar{S}$ usually forms at 1200–1250 °C [15], and begins to decompose higher than 1300 °C [14,16], while C_3_S starts to form in abundance higher than 1350 °C [17]. CaF_2_ [13,18,19] was used as mineralizer to reduce the viscosity of liquid phase and promote the formation of C_3_S by some scholars, in order to realize the coexistence of C_3_S and C_4_A_3_$\bar{S}$ in the same clinker system. However, this system still belongs to the Portland cement system (the content of C_3_S ranges from 30 wt.% to 50 wt.%), and it is easy to cause environmental pollution with Fluorine, so the use of CaF_2_ has been restricted in this field. In addition, no actual production has been reported for other methods [14,20]. It is found by our research group that at the same calcination temperature, the smaller the Al/Fe mole ratio of the clinker liquid phase is, the more favorable it is to reduce the formation temperature and viscosity of liquid phase, improving the burnability of raw materials and facilitating the formation of C_3_S [21]. The anhydrous barium calcium sulphoaluminate will be formed after calcining when barium is added into raw materials, and its decomposition temperature is higher than C_4_A_3_$\bar{S}$, which can reach above 1370 °C [22,23]. Based on the above research, the pilot production of alite (C_3_S) modified CSA (AMCSA) clinker was carried out by liquid phase manipulation and barium ion doping technology in Shandong Linqu Shengwei Special Cement Co. Ltd.

Calcium sulfate, as an important component of CSA, can be used to optimize setting time, strength development or volume stability, usually with an dosage of about 10–25 wt.% [24]. As shown in previous studies [5,25,26,27,28,29,30,31,32], the hydration products of CSA mainly depend on the dosage of calcium sulfate. In the presence of calcium sulfate, ettringite (AFt) forms together with aluminum hydroxide, and when the calcium sulfate is exhausted, monosulfate (AFm) and aluminum hydroxide are formed. In other words, the dosage of calcium sulfate can change the ratio of AFt to AFm in the hydration products [24]. Therefore, the effect of different gypsum dosage on setting time, hydration hardening and other properties of AMCSA were studied in order to determine the best proportion of the cement and provide practical experience and a theoretical basis for the production and application of the cement.

## 2. Raw Materials and Experimental Methods

### 2.1. Raw Materials

The raw materials used to calcine AMCSA clinker and prepare the cement are mainly limestone, bauxite, gypsum (dihydrate), barite and tailing sand, all of which are industry raw materials. Their chemical compositions are listed in Table 1, and they all come from Shandong Linqu Shengwei Special Cement Co. Ltd., Weifang, China.

### 2.2. Experimental Methods

#### 2.2.1. Preparation of AMCSA Clinker and Cement

The mineral composition design of AMCSA clinker is shown in Table 2. A certain amount of SO_3_ can improve the viscosity of liquid phase, increase the quantity of the liquid phase, benefit the formation of C_3_S, and stabilize the crystal form of C_3_S. However, excessive SO_3_ will inhibit the formation of C_3_S. Based on the optimal dosage of SO_3_ (about 2.0–3.0 wt.%) [33,34], the designed content of C_4_A_3_$\bar{S}$ is controlled at 21.7 wt.% through the mix proportion calculation and adjustment of raw materials in this study. Additionally, the barium ion was doped to design 24.3 wt.% C_3_BA_3_$\bar{S}$ with higher decomposition temperature. Based on the above conclusion that the smaller Al/Fe mole ratio in the liquid phase of clinker is, the more favorable it is for the formation of C_3_S, the iron phase was designed as C_2_F.

The AMCSA clinker was calcined in Shandong Linqu Shengwei Special Cement Co. Ltd.. Firstly, the mix proportion calculation was performed by the cumulative trial and error method. The raw materials were accurately weighed by a computer (Lenovo Group Ltd., Beijing, China) controlled automatic weighing system, and then homogenized by raw material mill (Shandong Guozhen Environmental Technology Equipment Co. Ltd., Weifang, China). The fineness of raw material was controlled to be 80 μm, and the sieve residue of square hole sieve was less than 5.0 wt.%. Then, raw materials were calcined on the production line with a daily production capacity of 500 tons of the company’s new-type dry-process predecomposition kiln (Xinxiang Great Wall machinery Co. Ltd., Xinxiang, China). The calcination temperature was controlled at 1320 °C to 1370 °C, the clinker litre weight which can reflect the mass of a certain number of particle size per unit volume was controlled about 1100 g/L, and the content of f-CaO was less than 0.2 wt.%. The clinker was cooled quickly in order to avoid mineral decomposition and crystal transformation of C_3_S.

The crushed clinker and the gypsum were ground to prepare cement by a planetary ball mill (Changsha Tencan Powder Technology Co. Ltd., Changsha, China) according to the mix ratio in Table 3. The cement was tested according to GB/T 8074-2008 *Testing method for specific surface of cement—Blaine method* and specific surface area was controlled at 360 ± 10 m^2^/kg.

#### 2.2.2. Test Method

**Setting time and compressive strength**. The setting time of AMCSA was measured according to GB/T1346-2011 *Test methods of water requirement of normal consistency, setting time and soundness of the Portland cements*. The compressive strength of AMCSA was tested in line with GB/T17671-1999 *Method of testing cement*—*Determination of strength*.

**Petrographic analysis**. Axio Scope A1 pol microscope (Carl Zeiss, Oberkochen, Germany) was adopted to observe the petrographic analysis of clinker in real time at the production site. The clinker specimens were placed into the forming mold by making fracture surface downward, and cold inlay materials (acrylic powder) were used to pour molding, and the molds were removed after curing. Then the clinker specimens were polished by using polishing powders with particle sizes of 80 μm, 30 μm, 10 μm and 1 μm respectively (from coarse to fine). The specimens were cleaned after each polishing with isopropanol in an ultrasonic cleaner (Shanghai Xin Yi Instruments and Meters Co. Ltd., Shanghai, China) for three times, 3 min each time. Finally, the specimens were observed after being etched with 1.0 wt.% of NH_4_Cl aqueous solution for 6 s (30 °C).

**XRD analysis and Rietveld refinement**. The XRD test was performed with the D8-ADVANCE X-ray diffractometer from Bruker, Billerica, MA, USA. The clinker minerals were quantitatively analyzed by using the method of Rietveld refinement. XRD full spectrum fitting was carried out based on the crystal structure of powder. The calculated and observed diffraction intensities were compared point by point by using a computer program, and the structural parameters and peak shape parameters were adjusted by the method of least square to make the calculated diffraction spectrum agree with the experimental spectrum, that was, the weighted residual factor R_wp_ was minimized [35].

**TG/DTG and SEM analysis**. TG/DTG analysis were tested by using Mettler-Toledo TGA/DSC 1 synchronous thermal analyzer (Mettler-Toledo, Columbus, OH, USA). The hydration heat release was measured by Eight-channel Thermometric TAM Air instrument (manufacturer, city, country). SEM was tested by ZEISS EVOLS15 scanning electron microscope (Carl Zeiss, Oberkochen, Germany).

## 3. Results and Analysis

### 3.1. Phase Analysis of AMCSA Clinker

**Figure 1:**
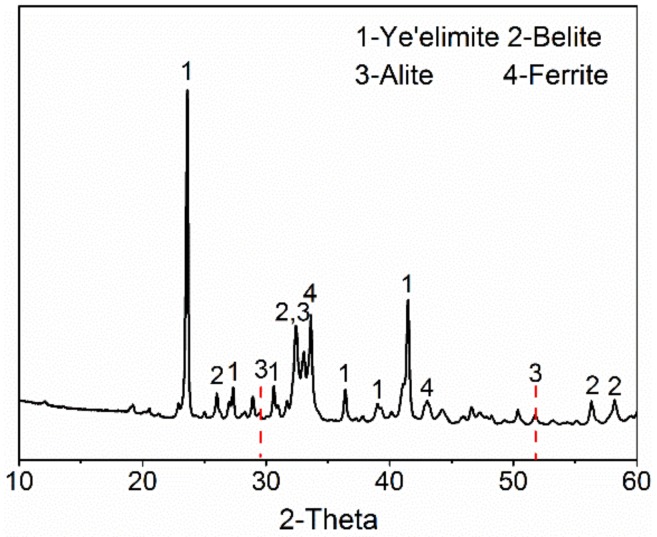
XRD pattern of AMCSA clinker.

The XRD pattern of AMCSA clinker is shown in Figure 1. It can be seen that the diffraction peaks’s intensity of the main mineral barium calcium sulphoaluminate (the sum of C_4_A_3_$\bar{S}$ and anhydrous barium calcium sulphoaluminate, hereinafter referred to as ye’elimite) and C_2_S are stronger. It proves that these two minerals of clinker grow well and they are abundant. The characteristic peaks of C_3_S are obviously observed in the pattern, which indicates that C_3_S is indeed generated in the clinker. Additionally, the characteristic diffraction peaks of C_4_A_3_$\bar{S}$ are shifted to the left, indicating the formation of anhydrous barium calcium sulphoaluminate mineral [22]. The characteristic diffraction peak of f-CaO (d = 2.40 Å) is not observed.

**Figure 2:**
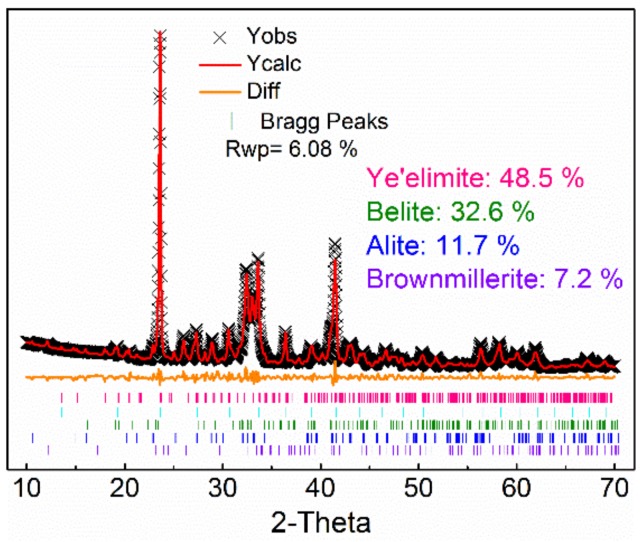
Quantitative analysis fitting of AMCSA clinker minerals.

In order to determine the minerals content of AMCSA clinker, its XRD pattern is quantitatively fitted by the Rietveld method, as shown in Figure 2. Better coincidence is shown in the fitted graph, the difference between the calculated graph and the experimental graph is low, and the R_wp_ is 6.08%, which indicates that the results are credible. The minerals content of ye’elimite, C_2_S, C_3_S and iron phase in the clinker are 48.5 wt.%, 32.6 wt.%, 11.7 wt.% and 7.2 wt.% respectively, which are close to the designed minerals composition, indicating that the clinker minerals are formed well and the coexistence of ye’elimite and C_3_S is achieved steadily in the clinker.

**Figure 3:**
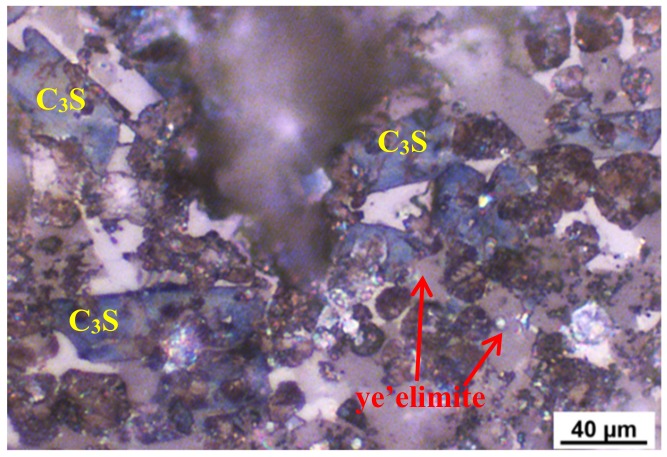
Petrographic picture of clinker observed at the production site.

The petrographic micrograph of the clinker of AMCSA is shown in Figure 3. It can be seen that, after being etched with 1.0 wt.% of NH_4_Cl, the shape of C_3_S is a long blue columnar, the size of C_3_S are about 5–40 μm, and the corrosion defects and blurred edges have appeared [36]. The shapes of C_2_S with uneven size are piles of brown ovoid or drops, and there are mineral inclusions inside the crystal and the corrosion defects are serious. Gray-black, regular morphology, a good degree of crystallization and neat edges can be seen in ye’elimite, presenting the hexagon shape reported in the literature [37]. The white iron phase is evenly filled in the other clinker minerals in an amorphous shape.

### 3.2. Effects of Gypsum on Setting Time of AMCSA

**Figure 4:**
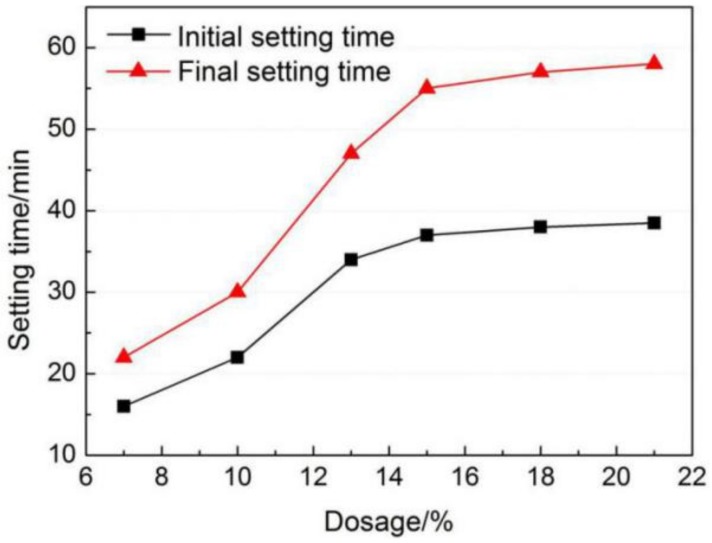
Effects of gypsum dosage on setting time of AMCSA.

It can be seen from Figure 4 that both the initial and final setting time of AMCSA are prolonged with the increasing gypsum dosage. When the gypsum dosage is less than 15 wt.%, the setting time increases significantly with the increasing gypsum dosage. The initial and final setting time increases from 16 min and 22 min to 37 min and 55 min respectively when the gypsum dosage increases from 7 wt.% to 15 wt.%. It can be noticed that the setting time of AMCSA increases very slowly with the increasing gypsum dosage when the gypsum dosage is higher than 15 wt.%. The results indicate that the setting time of AMCSA can be prolonged by adding gypsum, but the effect is not obvious when the gypsum dosage is higher than a certain amount.

### 3.3. Effects of Gypsum on Hydration Rate of AMCSA

**Figure 5:**
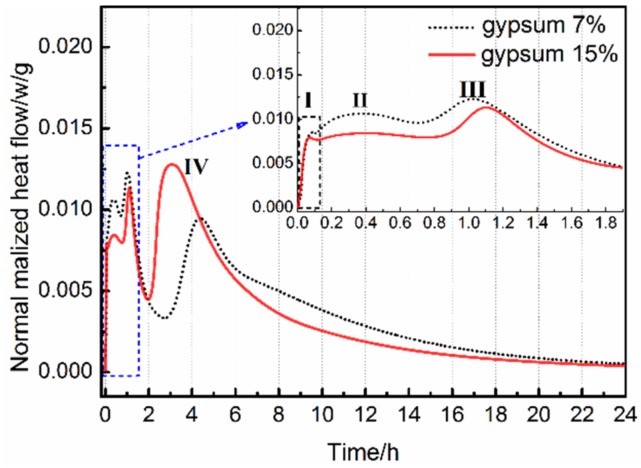
Effects of gypsum dosage on hydration of AMCSA.

The hydration rate of AMCSA with 7 wt.% and 15 wt.% gypsum dosages is shown in Figure 5. It can be seen that there are four exothermic peaks in the hydration rate curve of AMCSA. The first exothermic peak is caused due to heat released by dissolution when ye’elimite, C_3_S and gypsum are in contact with water. The second and third exothermic peaks are caused by the hydration of anhydrous barium calcium sulphoaluminate and C_4_A_3_$\bar{S}$ respectively. The hydration rate of anhydrous barium calcium sulphoaluminate is faster than C_4_A_3_$\bar{S}$ [22]. Part of the ye’elimite reacts with a small amount of Ca^2+^ and OH^−^, which are produced by the surface hydrolysis of gypsum and C_3_S particles, to form AFt (as Equation (1)), and part of the ye’elimite reacts with the gypsum to generate AFt and AH_3_(gel) (as Equation (2)). The fourth peak is a comprehensive exothermic peak. Because the content of C_3_S is low in clinker and the AH_3_(gel) produced by the hydration reaction of ye’elimite is sufficient, the C_3_S reacts with the AH_3_(gel) to form C_2_ASH_8_ and Ca(OH)_2_ (as Equation (3)) [12,38]. The Ca(OH)_2_ continues to hydrate with AH_3_(gel) and gypsum to generate AFt (as Equation (4)) [12,39]. The overall reaction process of the above four exothermic peaks is as follows:

(1) $$C_{4}A_{3}\bar{S}+8C\bar{S}H_{2}+6\mathrm{CH}+74H\to 3C_{6}A{\bar{S}}_{3}H_{32}$$

(2) $$C_{4}A_{3}\bar{S}+2C\bar{S}H_{2}+34H\to C_{6}A{\bar{S}}_{3}H_{32}+2{\mathrm{AH}}_{3}(\mathrm{gel})$$

(3) $$C_{3}S+{\mathrm{AH}}_{3}(\mathrm{gel})+6H\to C_{2}{\mathrm{ASH}}_{8}+\mathrm{CH}$$

(4) $$3\mathrm{CH}+{\mathrm{AH}}_{3}(\mathrm{gel})+3C\bar{S}H_{2}+16H\to C_{6}A{\bar{S}}_{3}H_{32}$$

Since the ye’elimite hydration rate is faster than C_3_S, the initial and final setting time of AMCSA are most related to the hydration of ye’elimite, that is, to the second and third exothermic peaks. It can be seen from Figure 5 that the setting time of AMCSA is delayed because the hydration rate of ye’elimite is slowed down by the increasing gypsum dosage. This is related to the fact that the denser AFt protective film is formed quickly on the surface of ye’elimite due to the addition of more gypsum, and then the contact of ye’elimite with water is hindered [40]. The retarding effect is not obvious if the gypsum dosage is increased after the gypsum dosage is sufficient, which is consistent with the experimental results shown in Figure 4. In addition, the hydration of C_3_S can be indirectly promoted by the gypsum according to the Equations (3) and (4). Thus the fourth exothermic peak is ahead of schedule due to the increasing gypsum dosage (as shown in Figure 5).

### 3.4. Effects of Gypsum on Compressive Strength of AMCSA Mortar

**Figure 6:**
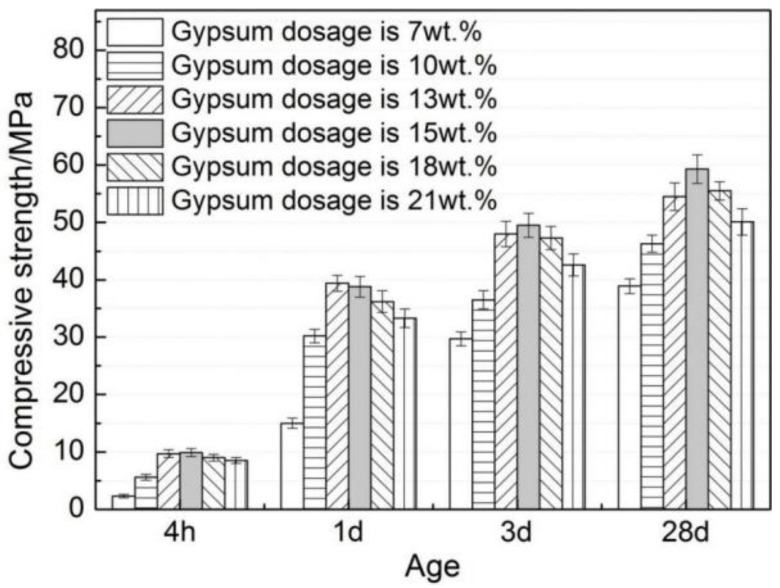
Effect of gypsum dosage on compressive strength of AMCSA mortar.

It can be seen from Figure 6 that an increasing trend is shown in the compressive strength of AMCSA mortar with the increase of age. The trend of increase first and then decrease is present at all ages of the compressive strength of AMCSA mortar with the increasing gypsum dosage. The highest value of the compressive strength of AMCSA mortar is found when the gypsum dosage is 15 wt.%, reaching up to 49.5 MPa at 3 days, and the compressive strength of AMCSA mortar at 28 days increases greatly compared with that at 3 days, increasing by 19.8%. This is different from CSA’s characteristics of quick hardening, high early strength and slow strength development in the middle and late stages. This is mainly caused by the role of C_3_S on the persistence of strength development in the middle and late stages of AMCSA mortar.

### 3.5. Effects of Gypsum on Hydration Products of AMCSA

#### 3.5.1. XRD Analysis

**Figure 7:**
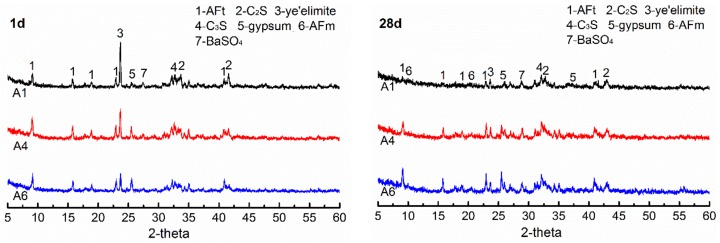
XRD patterns of AMCSA hydration for 1 d and 28 d.

The XRD analyses of the specimens A1, A4 and A6, at 1 day and at 28 days, are shown in Figure 7. The gypsum dosages in A1, A4 and A6 are 7 wt.%, 15 wt.% and 21 wt.%, respectively. It can be seen that the unreacted clinker minerals such as ye’elimite, C_3_S and C_2_S remain in different specimens at different ages, and their diffraction peak intensities decrease gradually with increasing hydration age, which indicates that the minerals in AMCSA are gradually hydrating. The diffraction peaks of AFt in hydration products of A4 and A6 are sharper than those of A1 at all ages. Especially at 28 days the diffraction peaks of AFt and gypsum in A1 essentially disappear and a large amount of AFt is transformed into AFm, which indicates that the gypsum dosage in A1 is insufficient. The AFt and gypsum diffraction peaks of A4 are still obvious at 28 days, and the gypsum diffraction peaks of A6 are sharper than those of A4. It further shows that the demand is met when the gypsum dosage is 15 wt.%, and the later strength development of AMCSA mortar is influenced by the excessive gypsum dosage in A6. In addition, the diffraction peaks of BaSO_4_ are seen in XRD patterns of different specimens at different ages due to the barium ions doping.

#### 3.5.2. TG/DTG Analysis

**Figure 8:**
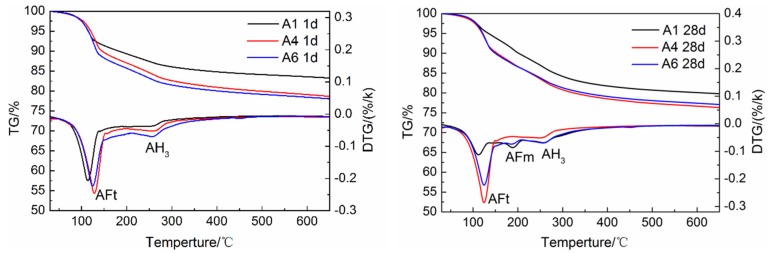
TG/DTG analysis of AMCSA hydration for 1 d and 28 d.

The TG/DTG curves of specimens A1, A4 and A6 at 1 day and 28 days are shown in Figure 8. It can be seen that AFt and AH_3_ are formed in the hydration products of each sample at the hydration age of 1 day, which is consistent with the above hydration rate and XRD analysis results. At the hydration age of 28 days, compared with the other two samples, the AFt endothermic peak of A1 decreases significantly, while the AFm endothermic peak is the most obvious, indicating that AFt is converted into AFm in large quantities, further confirming that the dosage of gypsum in A1 is insufficient. The AFm endothermic peaks of A4 and A6 are not obvious, which also confirms that the dosage of gypsum in A4 meets the requirements, which is consistent with other analysis results. Further observation shows that AH_3_ exothermic peaks of A4 and A6 are larger than that of A1 at 1 day, but smaller at 28 days, indicating that the AH_3_ content of A4 and A6 was reduced. This is because, as shown in Equations (1)–(4), the sufficient gypsum can promote the indirect mutual promotion of hydration between C_3_S and ye’elimite, the C_3_S reacts with the AH_3_ generated by ye’elimite to form C_2_ASH_8_ and Ca(OH)_2_. The Ca(OH)_2_ continues to hydrate with the AH_3_ and gypsum to generate AFt.

#### 3.5.3. SEM Analysis

**Figure 9:**
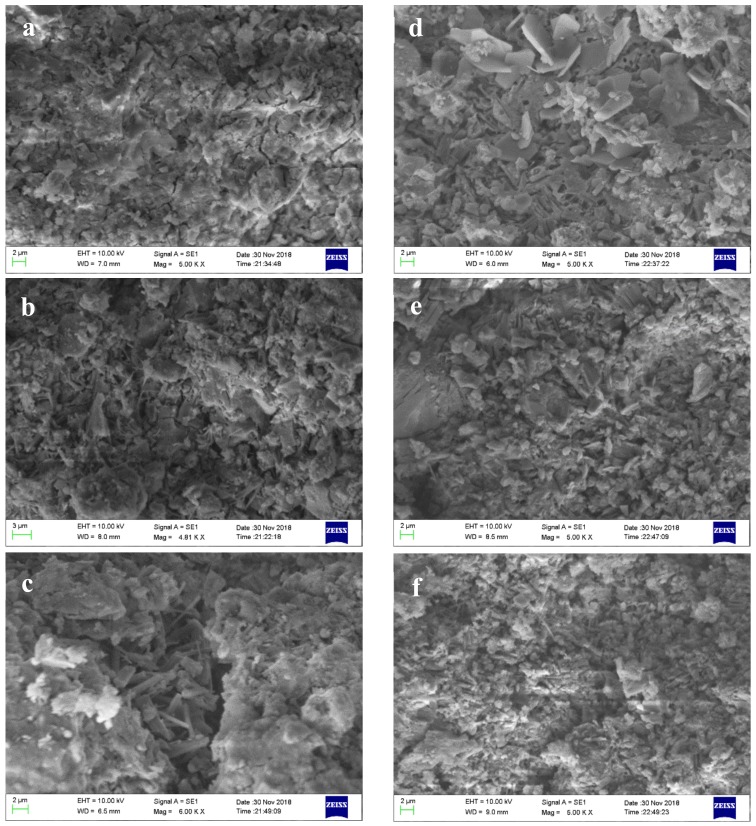
SEM pictures of AMCSA hydration for 1 day and 28 days. Note: (**a**–**c**) are SEM pictures of specimen A1, A4 and A6 at 1 day, and (**d**–**f**) are SEM pictures of specimens A1, A4 and A6 at 28 days respectively.

It can be seen from Figure 9 that the needle-like AFt and amorphous gels are produced in AMCSA at 1 day, and the amount of AFt produced by A4 and A6 is much higher than that from A1. The amount of AFt decreases, the gels increase, and the thin-sheet like AFm in A1 increases obviously after the hydration of 28 days in all specimens, proving again that the amount of gypsum in specimen A1 is insufficient. There is no obvious difference in amount of AFm between A4 and A6, which indicates that the 15 wt.% gypsum dosage can meet the demand. This is consistent with the experimental results obtained above.

## 4. Conclusions

(1) AMCSA clinker was calcined by liquid phase control and barium ion doping technology. The mineral content of ye’elimite, C_2_S, C_3_S and iron phase in AMCSA clinker are 48.5 wt.%, 32.6 wt.%, 11.7 wt.% and 7.2 wt.% respectively by Rietveld quantitative calculation, which are close to the designed mineral composition. The stable coexistence of ye’elimite and C_3_S in the same clinker system is realized.
(2) In a certain range of dosage under the experimental conditions in this study, the initial and final setting time of AMCSA are prolonged with the increasing gypsum dosage. The initial and final setting time increases from 16 min and 22 min to 37 min and 55 min respectively when the gypsum dosage increases from 7 wt.% to 15 wt.%. However, the setting time of AMCSA increases slowly with the increasing gypsum dosage after the gypsum dosage exceeds 15 wt.%.
(3) The trend of increasing first and then decreasing is present with the increasing gypsum dosage at all ages of the compressive strength of AMCSA mortar. The highest value of the compressive strength of AMCSA mortar is found when the gypsum dosage is 15 wt.%, and the strength of AMCSA mortar at 28 days still increases greatly compared with that at 3 days. The shortcoming of the low persistence of the strength growth in the middle and late stages of CSA is improved, which is mainly because of the role played by C_3_S.

## Figures and Tables

**Table 1:** Chemical composition of raw materials (wt.%).

Raw Materials	L.O.I	CaO	SiO_2_	Al_2_O_3_	Fe_2_O_3_	SO_3_	MgO	BaO
limestone	42.63	51.99	1.80	0.51	0.35	/	2.41	/
bauxite	16.01	2.05	16.92	58.58	2.70	/	0.24	/
gypsum	9.67	37.66	0.23	1.42	0.47	45.46	3.53	/
barite	6.45	1.09	2.98	0.88	0.24	27.27	0.39	59.56
tailing sand	2.53	4.00	60.69	3.95	23.06	/	4.20	/

**Table 2:** Mineral composition design of alite (C_3_S) modified CSA (AMCSA) clinker (wt.%).

$C_{4}A_{3}\bar{S}$	$C_{3}{\mathrm{BA}}_{3}\bar{S}$	C_3_S	C_2_S	C_2_F
21.7	24.3	10.0	35.0	7.0

**Table 3:** Dosage design of gypsum in cement (wt.%).

No.	Clinker Dosage	Gypsum Dosage
A1	93	7
A2	90	10
A3	87	13
A4	85	15
A5	82	18
A6	79	21
